# Supplementary material for: Heavy-atom tunnelling in singlet oxygen deactivation predicted by instanton theory with branch-point singularities
Source: Nat Commun. 2024 May 21;15:4335. doi: 10.1038/s41467-024-48463-2 (PMC11522392; doi:10.1038/s41467-024-48463-2)
Supplement: Supplementary file 1 — Supplementary Information [file 41467_2024_48463_MOESM1_ESM.pdf]

Supplementary information for: Heavy-atom tunnelling in singlet oxygen deactivation predicted by instanton theory with branch-point singularities

Imaad M. Ansari, Eric R. Heller, George Trenins and Jeremy O. Richardson

April 22, 2024

Supplementary Note 1: Harmonic oscillator flux correlation function

For the model system defined in the main text, the analytic expressions for the terms in c_{ff} are given in terms of the action

$$S(\mathbf{x}, z) = \frac{1}{2}\left(\mathbf{x}_1^{\mathrm{T}} \mathsf{A}_1 \mathbf{x}_1 + \mathbf{x}_2^{\mathrm{T}} \mathsf{A}_2 \mathbf{x}_2\right) - \mathbf{b}_1^{\mathrm{T}} \mathbf{x}_1 + c_1, \tag{1}$$

with

$$\mathbf{x}_i = \begin{pmatrix} x_i' & x_i'' \end{pmatrix}^{\mathrm{T}}, \; i \in \{1, 2\}, \tag{2a}$$

$$\mathsf{A}_1 = m\omega_1 \begin{pmatrix} t_1 & s_1 \\ s_1 & t_1 \end{pmatrix}, \tag{2b}$$

$$\mathsf{A}_2 = m\sqrt{\omega_{\mathrm{R}}\omega_{\mathrm{P}}} \begin{pmatrix} t_2 & s_2 \\ s_2 & t_2 \end{pmatrix}, \tag{2c}$$

$$t_1 = \frac{1}{\tanh(\omega_1(\beta\hbar - z))} + \frac{1}{\tanh(\omega_1 z)}, \tag{2d}$$

$$s_1 = -\frac{1}{\sinh(\omega_1(\beta\hbar - z))} - \frac{1}{\sinh(\omega_1 z)}, \tag{2e}$$

$$t_2 = \frac{\sqrt{\omega_{\mathrm{R}}/\omega_{\mathrm{P}}}}{\tanh(\omega_{\mathrm{R}}(\beta\hbar - z))} + \frac{\sqrt{\omega_{\mathrm{P}}/\omega_{\mathrm{R}}}}{\tanh(\omega_{\mathrm{P}} z)}, \tag{2f}$$

$$s_2 = -\frac{\sqrt{\omega_{\mathrm{R}}/\omega_{\mathrm{P}}}}{\sinh(\omega_{\mathrm{R}}(\beta\hbar - z))} - \frac{\sqrt{\omega_{\mathrm{P}}/\omega_{\mathrm{R}}}}{\sinh(\omega_{\mathrm{P}} z)}, \tag{2g}$$

$$\mathbf{b}_1 = m\zeta\omega_1 \tanh(\omega_1 z/2) \begin{pmatrix} 1 & 1 \end{pmatrix}^{\mathrm{T}}, \tag{2h}$$

$$c_1 = -\varepsilon z + m\zeta^2\omega_1 \tanh(\omega_1 z/2). \tag{2i}$$

The stationary point condition $\frac{\partial S}{\partial \mathbf{x}} = 0$ results in the following system of linear equations

$$\mathsf{A}_1 \mathbf{x}_1 = \mathbf{b}_1, \tag{3a}$$

$$\mathsf{A}_2 \mathbf{x}_2 = 0. \tag{3b}$$

$\mathsf{A}_1(\tau)$ is invertible for all τ and thus there exists a unique solution to Eq. (3a), i.e., $\tilde{\mathbf{x}}_1 = \mathsf{A}_1^{-1}\mathbf{b}_1$. At the branch point, $\det(\mathsf{A}_2) = 0$ and thus there exist infinitely many $\tilde{\mathbf{x}}_2$ which satisfy Eq. (3b). The general form of the solutions to Eq. (3b) can be written as $\tilde{x}_2' = -\frac{s_2}{t_2}\tilde{x}_2''$. If $\omega_{\mathrm{R}} > \omega_{\mathrm{P}}$, $t_2 - s_2 = 0$ can have a solution but not $t_2 + s_2 = 0$ and the infinite set of solutions are given by $\tilde{x}_2' = -\tilde{x}_2''$. If $\omega_{\mathrm{P}} > \omega_{\mathrm{R}}$, both $t_2 - s_2 = 0$ and $t_2 + s_2 = 0$ can have solutions when $|\omega_{\mathrm{P}}\tau_{\mathrm{P}}| > |\omega_{\mathrm{R}}\tau_{\mathrm{R}}|$ or $|\omega_{\mathrm{P}}\tau_{\mathrm{P}}| < |\omega_{\mathrm{R}}\tau_{\mathrm{R}}|$ respectively. The solution to the latter will have a smaller magnitude and will therefore correspond to the branch point closest to 0. The solutions for this case are given by $\tilde{x}_2' = +\tilde{x}_2''$, of which there are an infinite number.

The action for $\mathbf{x}$ that satisfies Eq. (3) is

$$\begin{aligned}\phi(z) = S(\mathbf{x}(z), z) &= c_1 - \frac{1}{2}\mathbf{b}_1^{\mathrm{T}}\mathsf{A}_1^{-1}\mathbf{b}_1 \\ &= -\varepsilon z + m\zeta^2\omega_1 \frac{\sinh(\omega_1(\beta\hbar - z)/2)\sinh(\omega_1 z/2)}{\sinh(\omega_1\beta\hbar/2)}.\end{aligned} \tag{4}$$

The terms in the prefactor of c_{ff} are

$$C_{\mathrm{R}}(z) = \frac{m^2\omega_1\omega_{\mathrm{R}}}{\sinh(\omega_1(\beta\hbar - z))\sinh(\omega_{\mathrm{R}}(\beta\hbar - z))}, \tag{5a}$$

$$C_{\mathrm{P}}(z) = \frac{m^2\omega_1\omega_{\mathrm{P}}}{\sinh(\omega_1 z)\sinh(\omega_{\mathrm{P}} z)}, \tag{5b}$$

$$C(z) = \det(\mathsf{A}_1)\det(\mathsf{A}_2), \tag{5c}$$

$$\begin{aligned}\Theta(z) = \frac{C(z)}{C_{\mathrm{R}}(z)C_{\mathrm{P}}(z)} &= \left(\frac{1}{Z_{\mathrm{R}}^{(1)}}\right)^2 \Big\{2[\cosh(\omega_{\mathrm{R}}(\beta\hbar - z))\cosh(\omega_{\mathrm{P}} z) - 1] \\ &+ \left(\frac{\omega_{\mathrm{P}}}{\omega_{\mathrm{R}}} + \frac{\omega_{\mathrm{R}}}{\omega_{\mathrm{P}}}\right)\sinh(\omega_{\mathrm{R}}(\beta\hbar - z))\sinh(\omega_{\mathrm{P}} z)\Big\},\end{aligned} \tag{5d}$$

where $Z_{\mathrm{R}}^{(1)}$ is the contribution from the reaction coordinate $\mathbf{x}_1$ to the reactant partition function

$$Z_{\mathrm{R}}^{(1)} = \frac{1}{2\sinh(\beta\hbar\omega_1/2)}. \tag{6}$$

Supplementary Note 2: Data for on-the-fly *ab-initio* calculations

Figure 1 compares the MCSCF PESs along the branch-point instanton to the MRMP2 PESs along the same path. This is a test of the effect of dynamic correlation on the reaction. Apart from an overall shift, the two sets of PESs exhibit small differences in curvature, which will slightly shift the location of the branch point and thus affect the corresponding instanton rate. However, predicting whether this will increase or decrease the rate is not trivial and is left for a future study.

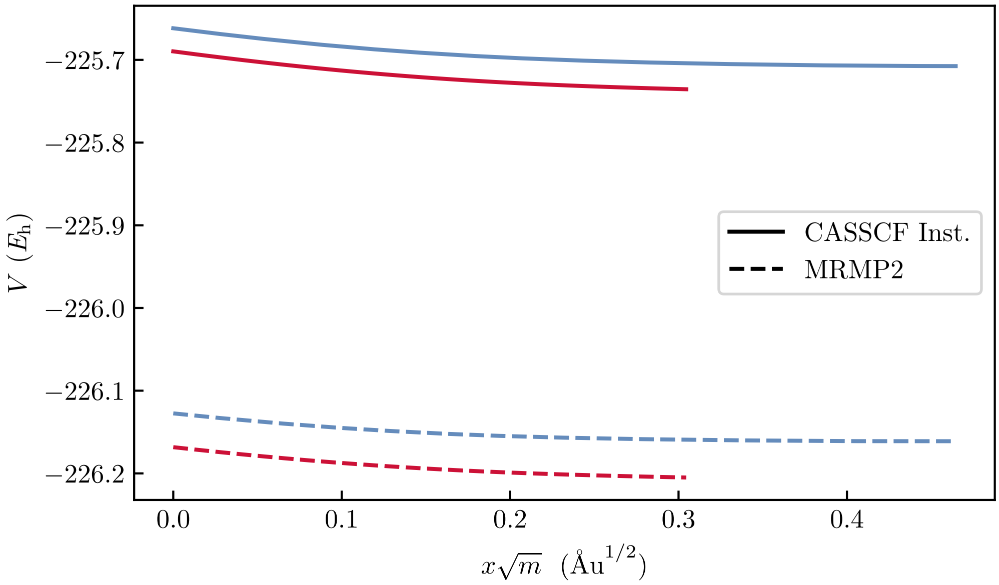

Supplementary Figure 1: The PES along the branch-point instanton for $O_2 \cdots H_2O$ at 300 K calculated using MCSCF (solid lines), compared to the PES along the same path calculated with MRMP2 (dashed lines). The x-axis is the mass-weighted path length. Blue corresponds to the reactant and red to the product. Source data are provided as a Source Data file.

2.1 Vibrational Analysis

Table 1 shows the contributions of translations (Γ_{trans}), rotations (Γ_{rot}) and vibrations (Γ_{vib}) to the trace of the reducible representation of 15-dimensional Cartesian coordinates ($\Gamma_{3\mathrm{N}}$) in the $C_{2\mathrm{v}}$ point group. The vibrational contribution can be further expressed as a sum of irreducible representations, $\Gamma_{\mathrm{vib}} = 4A_1 + A_2 + B_1 + 2B_2$. The normal modes of the reactant minimum and the product saddle point (both with $C_{2\mathrm{v}}$ point group) are shown in Supplementary Figures 2 and 3, with the corresponding frequencies in Supplementary Table 2.

Supplementary Table 1: Character table for the reducible representations of translations, rotations and vibrations for a system with $C_{2\mathrm{v}}$ symmetry and 15 degrees of freedom.

	E	$C_\mathrm{v}(\mathrm{z})$	$\sigma_\mathrm{v}(\mathrm{xz})$	$\sigma_\mathrm{v}(\mathrm{yz})$
$\Gamma_{3\mathrm{N}}$	15	−1	3	3
Γ_{trans}	3	−1	1	1
Γ_{rot}	3	−1	−1	−1
Γ_{vib}	9	1	3	3

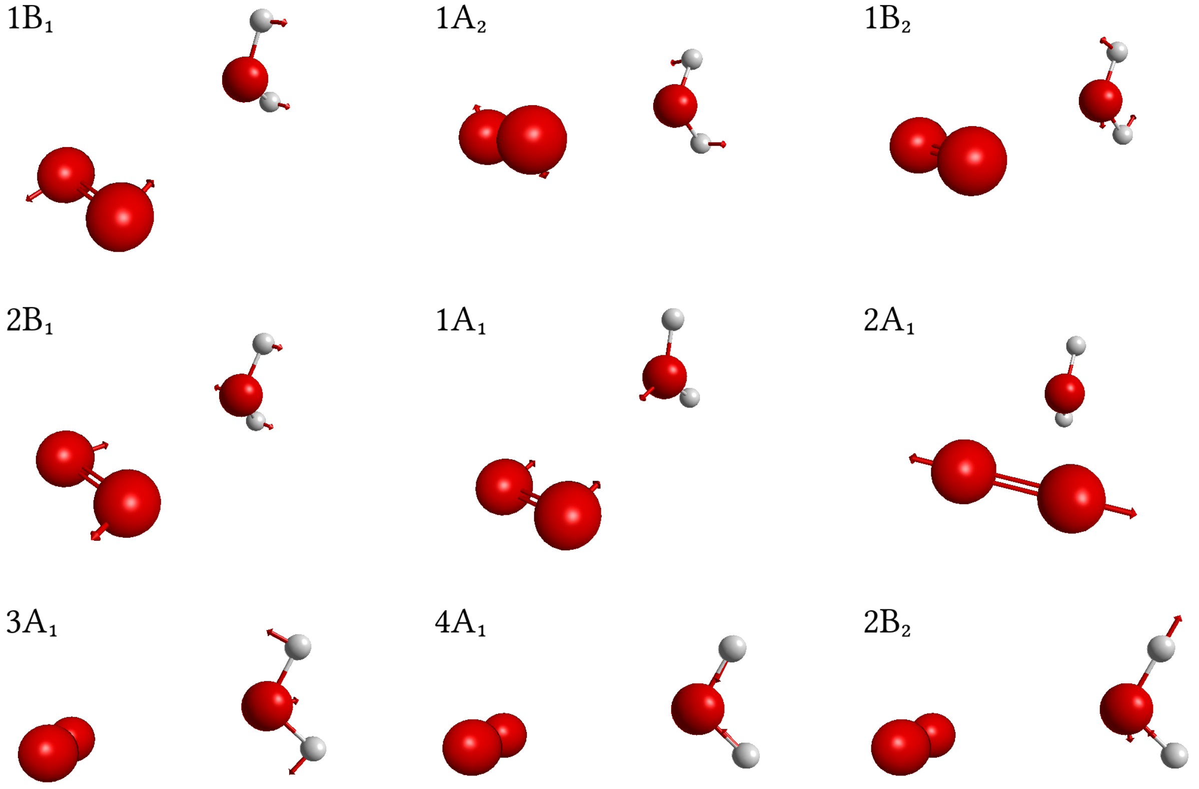

Supplementary Figure 2: Normal modes of the $O_2 \cdots H_2O$ reactant minimum with C_{2v} symmetry. To assign irreducible representations to the normal modes, the axes were chosen such that the z-axis passes through the oxygen atom of H_2O and bisects the O_2 bond, the y-axis is in the H_2O plane and the x-axis is perpendicular to this plane.

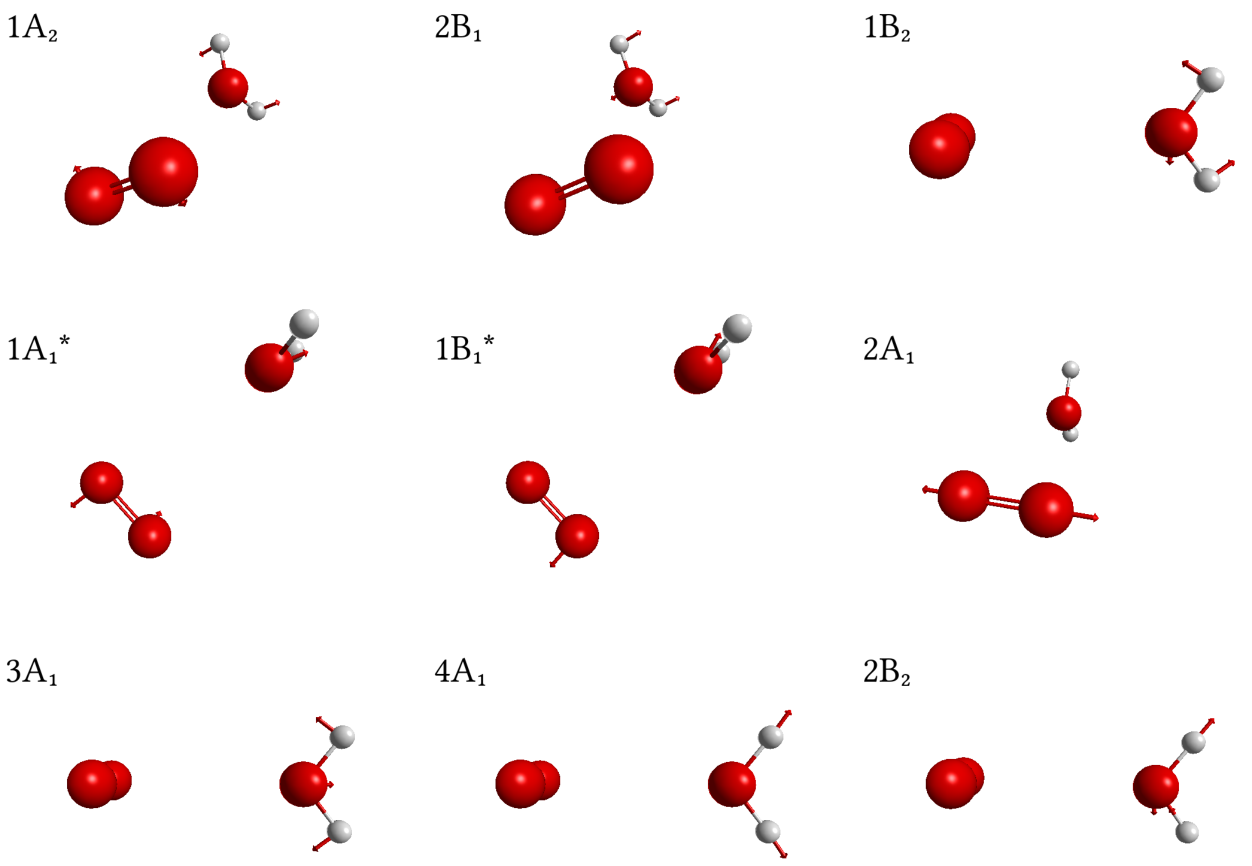

Supplementary Figure 3: Normal modes of the $O_2 \cdots H_2O$ product saddle point (with two imaginary modes) with C_{2v} symmetry. The modes labelled A_1^* and $1B_1^*$ are accidental near-degenerate modes. A linear combination $1A_1^* + 1B_1^*$ gives a mode with A_1 symmetry while $1A_1^* - 1B_1^*$ results in a mode that has B_1 symmetry.

Supplementary Table 2: Frequencies at minima and the second-order product saddle point with $C_{2\mathrm{v}}$ symmetry.

System	Configuration	Frequencies (cm^{-1})								
		$1B_1$	$1A_2$	$1B_2$	$2B_1$	$1A_1$	$2A_1$	$3A_1$	$4A_1$	$2B_2$
$O_2\cdots H_2O$	Reactant min.	39.8	50.2	54.2	72.7	83.4	1428.8	1775.0	4114.5	4213.1
	Product sad. (2)	54.6*	i42.0	31.0	i34.2	54.6*	1532.5	1775.5	4114.0	4212.5
$O_2\cdots D_2O$	Reactant min.	32.3	37.2	40.0	67.5	80.7	1428.8	1298.7	2967.5	3087.3
$^{18}O_2\cdots H_2O$	Reactant min.	38.6	49.9	54.2	70.7	81.7	1346.9	1775.0	4114.5	4213.1

Two normal modes of the product saddle point were found to be nearly degenerate with frequencies of $53.7\,\mathrm{cm}^{-1}$ and $55.5\,\mathrm{cm}^{-1}$ and are indicated by a star next to the frequency values and on the label in Supplementary Figure 3. A sum of the two modes results in a mode with A_1 while a difference gives a mode with B_1 symmetry. The reported frequency is the average of the two. Geometries, masses and Hessians are available as a Source Data file.

2.2 NA-TST

The non-adiabatic transition-state theory (NA-TST) rate constant is given by [1]

$$k_{\text{NA-TST}} = \frac{|\Delta|^2}{\hbar|\boldsymbol{\kappa}_\text{R} - \boldsymbol{\kappa}_\text{P}|}\sqrt{\frac{2\pi}{\beta\hbar^2}}\frac{Z_\text{MECP}}{Z_\text{R}}\mathrm{e}^{-\beta V^\ddagger}, \tag{7}$$

where $\boldsymbol{\kappa}_\text{R}$ and $\boldsymbol{\kappa}_\text{P}$ are the mass-weighted gradients on the reactant and product PESs surfaces at the MECP, Δ is the spin–orbit coupling at the MECP geometry, $V^\ddagger$ is the barrier height of the MECP relative to the reactant minimum, and Z_MECP and Z_R are the partition functions of the MECP and the reactant minimum, expressed as the product of translational, rotational and vibrational contributions

$$Z_\text{MECP} = Z^\text{trans}_\text{MECP} Z^\text{rot}_\text{MECP} Z^\text{vib}_\text{MECP}, \tag{8a}$$

$$Z_\text{R} = Z^\text{trans}_\text{R} Z^\text{rot}_\text{R} Z^\text{vib}_\text{R}. \tag{8b}$$

The translational and rotational contributions are given by the usual expressions for the free-particle and rigid-rotor partition functions [1]. The rotational partition function includes the symmetry number, which is equal to the size of the rotational subgroup. As the reactant minimum has $C_{2\text{v}}$ symmetry and the MECP has C_s symmetry, the resulting ratio of symmetry numbers is equal to 2. The translational partition functions cancel exactly.

The quantum harmonic oscillator partition function was used for the vibrational contribution to the reactant partition function. The frequencies at the reactant minimum after projecting translations and rotations are listed in Supplementary Table 2. For the MECP, an effective Hessian was constructed as $\boldsymbol{H}_\text{MECP} = (1-\lambda)\boldsymbol{H}_\text{R} + \lambda\boldsymbol{H}_\text{P}$, where $\boldsymbol{H}_\text{R}$ and $\boldsymbol{H}_\text{P}$ are the Hessians at the MECP geometry on the reactant and product PESs respectively and λ is the Lagrange multiplier at the MECP, defined as $\lambda = -||\boldsymbol{\kappa}_\text{R}||/||\boldsymbol{\kappa}_\text{R} - \boldsymbol{\kappa}_\text{P}||$. $\boldsymbol{H}_\text{MECP}$ is subsequently mass-weighted and the translational, rotational and reactive modes are projected out, where the reactive mode is defined as that along $\boldsymbol{\kappa}_\text{R} - \boldsymbol{\kappa}_\text{P}$. The resulting matrix is diagonalized and the frequencies listed in Supplementary Table 3. The NA-TST rates at various temperatures are tabulated in Supplementary Table 4.

Supplementary Table 3: The frequencies of the effective Hessian at the MECP. The optimized value of the Lagrange multiplier λ is -1.991.

System	Frequencies (cm^{-1})							
$O_2 \cdots H_2O$	251.2	466.1	711.3	713.3	1078.1	1737.6	4076.3	4210.0
$O_2 \cdots D_2O$	191.6	416.9	519.1	585.9	1077.3	1272.5	2936.2	3090.4
$^{18}O_2 \cdots H_2O$	249.1	449.8	707.8	710.3	1023.2	1737.4	4074.8	4210.0

Supplementary Table 4: NA-TST rates. The values of Δ and $V^\ddagger$ used are $98.40\,\mathrm{cm^{-1}}$ and $1.796\,\mathrm{eV}$ respectively. The value of $|\boldsymbol{\kappa}_\mathrm{R} - \boldsymbol{\kappa}_\mathrm{P}|$ is $1.116\,\mathrm{eV\AA^{-1}u^{-1}}$, $1.107\,\mathrm{eV\AA^{-1}u^{-1}}$ and $1.079\,\mathrm{eV\AA^{-1}u^{-1}}$ for $O_2\cdots H_2O$, $O_2\cdots D_2O$ and $^{18}O_2\cdots H_2O$ respectively.

System	T (K)	$Z^\mathrm{rot}_\mathrm{MECP}/Z^\mathrm{rot}_\mathrm{R}$	$Z^\mathrm{vib}_\mathrm{MECP}/Z^\mathrm{vib}_\mathrm{R}$	$\mathrm{e}^{-\beta V^\ddagger}$	$k_\text{NA-TST}$ ($\mathrm{s^{-1}}$)
$O_2\cdots H_2O$	275	0.860	4.701×10^{-5}	1.233×10^{-33}	6.025×10^{-26}
	280	0.860	4.728×10^{-5}	4.771×10^{-33}	2.366×10^{-25}
	290	0.860	4.770×10^{-5}	6.210×10^{-32}	3.163×10^{-24}
	300	0.860	4.795×10^{-5}	6.813×10^{-31}	3.547×10^{-23}
	310	0.860	4.805×10^{-5}	6.404×10^{-30}	3.397×10^{-22}
	320	0.860	4.803×10^{-5}	5.233×10^{-29}	2.819×10^{-21}
	330	0.860	4.791×10^{-5}	3.765×10^{-28}	2.054×10^{-20}
$O_2\cdots D_2O$	280	0.879	6.795×10^{-5}	4.769×10^{-33}	3.503×10^{-25}
	290	0.879	6.687×10^{-5}	6.208×10^{-32}	4.566×10^{-24}
	300	0.879	6.571×10^{-5}	6.810×10^{-31}	5.007×10^{-23}
	310	0.879	6.451×10^{-5}	6.402×10^{-30}	4.697×10^{-22}
	320	0.879	6.327×10^{-5}	5.231×10^{-29}	3.824×10^{-21}
	330	0.879	6.201×10^{-5}	3.764×10^{-28}	2.739×10^{-20}
$^{18}O_2\cdots H_2O$	280	0.859	4.402×10^{-5}	4.771×10^{-33}	2.279×10^{-25}
	290	0.859	4.445×10^{-5}	6.210×10^{-32}	3.048×10^{-24}
	300	0.859	4.472×10^{-5}	6.813×10^{-31}	3.421×10^{-23}
	310	0.859	4.486×10^{-5}	6.404×10^{-30}	3.279×10^{-22}
	320	0.859	4.488×10^{-5}	5.233×10^{-29}	2.724×10^{-21}
	330	0.859	4.480×10^{-5}	3.765×10^{-28}	1.987×10^{-20}

Supplementary Table 5: Stationary-action points at 300 K.

System	N	$\tau_{\mathrm{SA}}/\beta\hbar$	$\phi_{\mathrm{SA}}/\hbar$
$O_2\cdots H_2O$	256	−0.783	21.053
	512	−0.785	21.030
$O_2\cdots D_2O$	256	−0.788	21.094
	512	−0.791	21.063

2.3 Instanton calculations

An example of the spline-fitting procedure for $O_2\cdots H_2O$ with 512 beads is given in Supplementary Figures 4 and 5 at 300 K.

At 300 K, a second branch point was found for $O_2\cdots H_2O$ further to the right of the stationary-action point (see Supplementary Table 5), at $\tau/\beta\hbar = -0.51$. In principle, a uniform approximation could be developed to explicitly include the effect of this branch point. However, as the action at this point was about $2\hbar$ larger than at the first branch point (at $\tau/\beta\hbar = -0.44$), it is clear that the effect of the second branch point is subdominant and can be neglected within a reasonable approximation.

In Supplementary Table 6, we present data for the branch-point instantons at 300 K. The rate is expressed in terms of ring-polymer partition functions

$$k_{\mathrm{BP}} = \frac{|\Delta|^2}{\hbar^2}\beta\hbar\frac{Z_{\mathrm{inst}}^{\mathrm{rot}}Z_{\mathrm{inst}}^{\mathrm{vib}}}{Z_{\mathrm{R}}^{\mathrm{rot}}Z_{\mathrm{R}}^{\mathrm{vib}}}\,\mathrm{e}^{-\phi_{\mathrm{BP}}/\hbar}, \tag{9}$$

where $Z_{\mathrm{inst}}^{\mathrm{vib}}$ is the vibrational partition function of the branch-point instanton defined as

$$Z_{\mathrm{inst}}^{\mathrm{vib}} = \sqrt{\frac{4\pi}{(\beta\mathcal{E}_{\mathrm{BP}})(\beta\hbar\Omega_{\mathrm{BP}})}}\,. \tag{10}$$

Similarly in Supplementary Table 7, we present data for the stationary-flux instantons, where the rate is expressed as

$$k_{\mathrm{SF}} = \sqrt{2\pi}\frac{|\Delta|^2}{\hbar^2}\beta\hbar\frac{Z_{\mathrm{inst}}^{\mathrm{rot}}Z_{\mathrm{inst}}^{\mathrm{vib}}}{Z_{\mathrm{R}}^{\mathrm{rot}}Z_{\mathrm{R}}^{\mathrm{vib}}}\,\mathrm{e}^{-\phi(\tau_{\mathrm{SF}})/\hbar}, \tag{11}$$

with

$$Z_{\mathrm{inst}}^{\mathrm{vib}} = \sqrt{\frac{1}{\mu_{\mathrm{SF}}\beta^2\hbar\Theta(\tau_{\mathrm{SF}})}}\,. \tag{12}$$

The rotational partition function of the ring-polymer $Z_{\mathrm{inst}}^{\mathrm{rot}}$ is the classical rigid-rotor partition function of the ring polymer [1, 2]. The expressions for the rotational and vibrational contributions to the reactant partition functions $Z_{\mathrm{R}}^{\mathrm{rot}}$ and $Z_{\mathrm{R}}^{\mathrm{vib}}$ are given by the usual rigid-rotor and quantum harmonic oscillator approximations respectively.

Branch-point rate constants between 275 K and 330 K for $O_2\cdots H_2O$ and between 280 K and 330 K for $O_2\cdots D_2O$ are presented in Supplementary Table 8 and plotted in Supplementary Figure 6. We do not present results for higher temperatures as there the effect of the second branch point is no longer negligible.

We note that the $O_2\cdots H_2O$ rate constant seems to plateau at low temperature, an indication of deep tunnelling. However, due to the manner in which the branch point is located and the numerical derivatives obtained, there is some noise in the data in this regime, which can be used to give an estimate of the numerical error in this method. Note that this error is not statistical, as instanton theory involves no sampling, but arises from numerous factors such as the convergence cutoff for the ring-polymer optimization and the location of the knots used to fit the spline. The standard deviation of the $T \le 310$ K rates for $O_2\cdots H_2O$ is found to be $\sigma^{\mathrm{H}} = 66.8\,\mathrm{s}^{-1}$, with a mean of $\mu^{\mathrm{H}} = 21\,282\,\mathrm{s}^{-1}$. We plot error bars of $\pm 2\sigma^{\mathrm{H}}$ to indicate the numerical errors for k_{BP} of $O_2\cdots H_2O$. The corresponding error bars for $O_2\cdots D_2O$, $\pm 2\sigma^{\mathrm{D}}$, are obtained by assuming that the relative error for $O_2\cdots D_2O$ is the same as that for $O_2\cdots H_2O$, i.e., $\sigma^{\mathrm{D}}(T)/k_{\mathrm{BP}}^{\mathrm{D}}(T) = \sigma^{\mathrm{H}}/\mu^{\mathrm{H}}$. Using this, we can estimate the numerical error for the kinetic isotope effect $k_{\mathrm{BP}}^{\mathrm{H}}(T)/k_{\mathrm{BP}}^{\mathrm{D}}(T)$ as $\pm 2|k_{\mathrm{BP}}^{\mathrm{H}}(T)/k_{\mathrm{BP}}^{\mathrm{D}}(T)|\sqrt{\left(\sigma^{\mathrm{H}}/k_{\mathrm{BP}}^{\mathrm{H}}(T)\right)^2 + \left(\sigma^{\mathrm{D}}/k_{\mathrm{BP}}^{\mathrm{D}}(T)\right)^2}$.

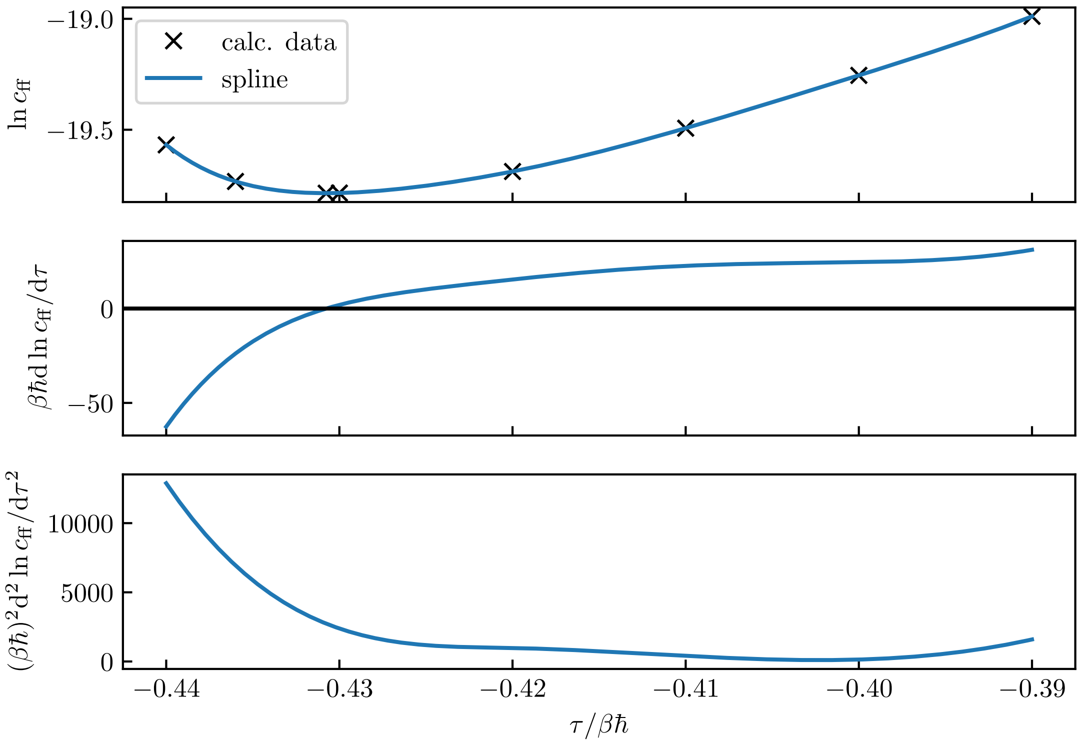

Supplementary Figure 4: The spline fit of $\ln c_{\rm ff}(\tau)$ used to find $\tau_{\rm SF}$ and calculate $\mu_{\rm SF}$. The data plotted corresponds to $O_2 \cdots H_2O$ with 512 beads at 300 K. Source data are provided as a Source Data file.

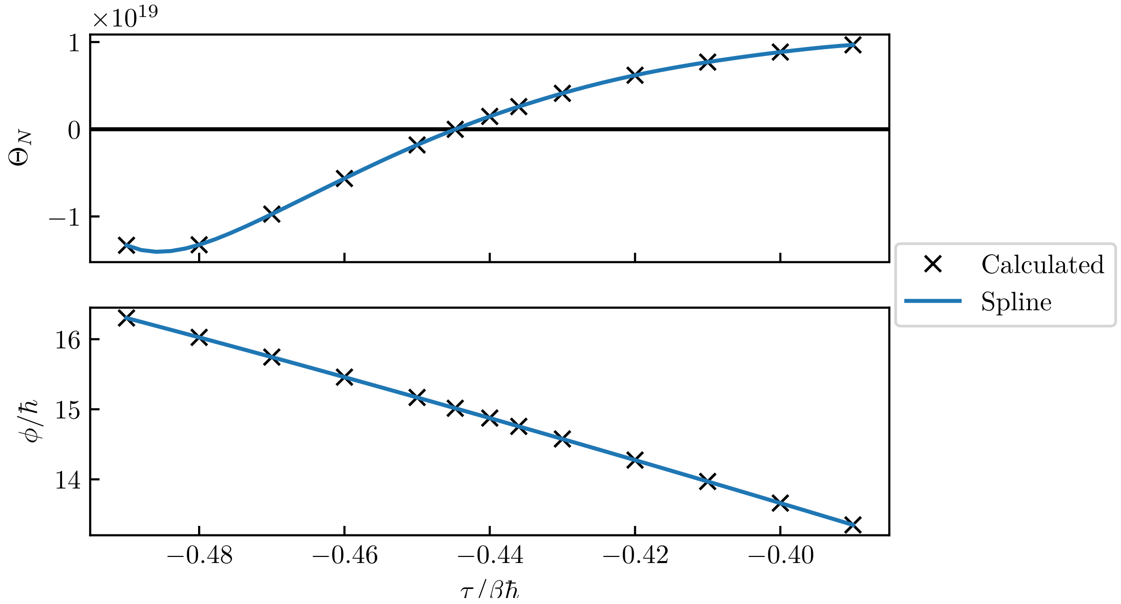

Supplementary Figure 5: The spline fit of $\Theta(\tau)$, used to find $\tau_{\rm BP}$ and calculate $\Omega_{\rm BP}$, and $\phi(\tau)$, used to calculate $\mathcal{E}_{\rm BP}$. The data plotted corresponds to $O_2 \cdots H_2O$ with 512 beads at 300 K. Source data are provided as a Source Data file.

Supplementary Table 6: Instanton calculations of singlet to triplet rates at the branch point τ_{BP} at 300 K.

El. struct.	System	N	$\tau_{BP}/\beta\hbar$	$\phi_{BP}/\hbar$	$\beta\mathcal{E}_{BP}$	$\beta\hbar\Omega_{BP}$	$Z^{rot}_{inst}/Z^{rot}_{R}$	$Z^{vib}_{inst}/Z^{vib}_{R}$	Δ (cm^{-1})	k_{BP} (s^{-1})
cc-pVDZ	$O_2\cdots H_2O$	256	−0.445	15.016	29.400	3.222×10^{20}	0.988	0.063	35.61	2.147×10^{4}
		512	−0.445	15.015	29.432	3.271×10^{20}	0.988	0.062	35.61	2.132×10^{4}
	$O_2\cdots D_2O$	256	−0.611	19.175	19.278	6.288×10^{13}	1.013	0.096	49.92	1.027×10^{3}
		512	−0.611	19.176	19.292	6.389×10^{13}	1.013	0.095	49.93	1.017×10^{3}
	$^{18}O_2\cdots H_2O$	256	−0.445	15.118	30.313	1.844×10^{20}	0.985	0.062	34.54	1.785×10^{4}
		512	−0.445	15.117	30.343	1.868×10^{20}	0.985	0.061	34.54	1.774×10^{4}
cc-pVTZ	$O_2\cdots H_2O$	256	−0.469	15.361	28.004	1.501×10^{20}	0.980	0.119	40.15	3.611×10^{4}
		508	−0.470	15.403	28.058	1.519×10^{20}	0.981	0.118	40.26	3.458×10^{4}
		764	−0.470	15.393	28.083	1.545×10^{20}	0.981	0.117	40.23	3.458×10^{4}
cc-pVDZ +PCM	$O_2\cdots H_2O$	256	−0.466	15.487	28.099	8.824×10^{23}	0.999	0.088	35.96	1.937×10^{4}
		512	−0.466	15.488	28.067	9.098×10^{23}	0.999	0.087	35.95	1.906×10^{4}

Supplementary Table 7: Instanton calculations of singlet to triplet rates at the stationary-flux point τ_{SF} at 300 K.

El. struct.	System	N	$\tau_{SF}/\beta\hbar$	$\phi/\hbar$	$\beta^2\hbar\mu_{SF}$	$\ln c_{ff}(\tau_{SF})$	$Z^{rot}_{inst}/Z^{rot}_{R}$	$Z^{vib}_{inst}/Z^{vib}_{R}$	Δ (cm^{-1})	k_{SF} (s^{-1})
cc-pVDZ	$O_2\cdots H_2O$	256	−0.431	14.611	2168.952	−19.759	0.987	0.019	34.85	2.382×10^{4}
		512	−0.431	14.599	2734.715	−19.786	0.987	0.017	34.83	2.062×10^{4}
	$O_2\cdots D_2O$	256	−0.592	18.791	1279.604	−16.220	1.009	0.025	47.55	8.827×10^{2}
		512	−0.591	18.785	1291.481	−16.225	1.009	0.024	47.50	8.727×10^{2}
	$^{18}O_2\cdots H_2O$	256	−0.432	14.713	2579.423	−19.362	0.984	0.018	33.92	1.873×10^{4}
		512	−0.431	14.695	3044.550	−19.389	0.984	0.016	33.90	1.677×10^{4}
cc-pVTZ	$O_2\cdots H_2O$	256	−0.454	14.949	2670.722	−19.783	0.979	0.028	39.02	3.001×10^{4}
		508	−0.455	14.976	5068.831	−19.822	0.979	0.020	39.09	2.113×10^{4}
cc-pVDZ +PCM	$O_2\cdots H_2O$	256	−0.449	14.985	1874.921	−24.200	0.996	0.026	34.96	2.195×10^{4}

Supplementary Table 8: Branch-point instanton rates as a function of temperature.

System	T (K)	N	$\tau_{BP}/\beta\hbar$	$\phi_{BP}/\hbar$	$\beta\mathcal{E}_{BP}$	$\beta\hbar\Omega_{BP}$	$Z^{rot}_{inst}/Z^{rot}_{R}$	$Z^{vib}_{inst}/Z^{vib}_{R}$	Δ (cm^{-1})	k_{BP} (s^{-1})
$O_2\cdots H_2O$	275	512	−0.408	15.034	32.101	1.239×10^{23}	0.991	0.059	35.40	2.130×10^{4}
	280	512	−0.415	15.029	31.525	3.483×10^{22}	0.990	0.060	35.44	2.136×10^{4}
	290	512	−0.430	15.030	30.435	3.113×10^{21}	0.989	0.061	35.54	2.126×10^{4}
	300	512	−0.445	15.015	29.432	3.271×10^{20}	0.988	0.062	35.61	2.132×10^{4}
	310	512	−0.459	15.006	28.474	3.973×10^{19}	0.987	0.063	35.69	2.117×10^{4}
	320	512	−0.474	14.999	27.587	5.234×10^{18}	0.986	0.065	35.77	2.142×10^{4}
	330	512	−0.488	14.990	26.747	7.751×10^{17}	0.985	0.067	35.85	2.166×10^{4}
$O_2\cdots D_2O$	280	512	−0.571	19.198	20.652	2.868×10^{15}	1.015	0.087	49.69	9.732×10^{2}
	290	512	−0.591	19.189	19.942	3.999×10^{14}	1.014	0.092	49.82	9.987×10^{2}
	300	512	−0.611	19.176	19.292	6.389×10^{13}	1.013	0.095	49.93	1.017×10^{3}
	310	512	−0.631	19.165	18.687	1.051×10^{13}	1.012	0.102	50.04	1.075×10^{3}
	320	512	−0.651	19.154	18.110	1.909×10^{12}	1.010	0.110	50.16	1.135×10^{3}
		768	−0.651	19.153	18.117	1.911×10^{12}	1.011	0.110	50.16	1.135×10^{3}
	330	512	−0.671	19.143	17.571	3.674×10^{11}	1.009	0.119	50.28	1.216×10^{3}

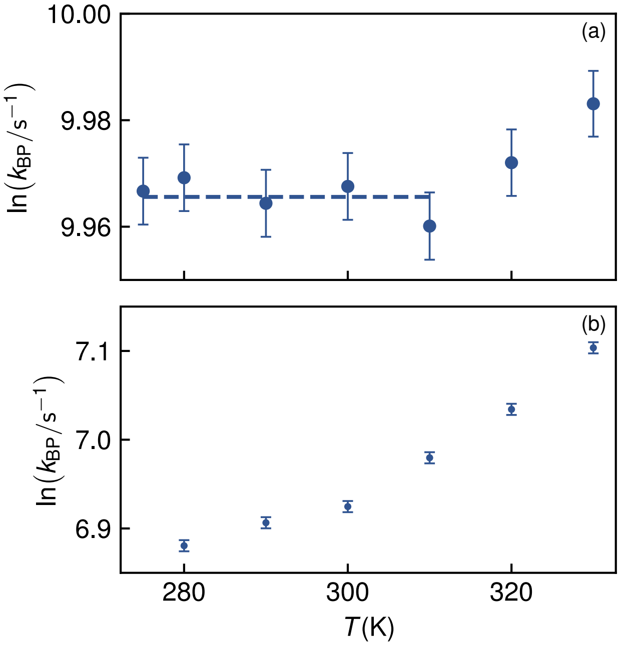

Supplementary Figure 6: Branch-point instanton rate constants as a function of temperature for (a) $O_2{\cdots}H_2O$ and (b) $O_2{\cdots}D_2O$. The procedure for obtaining the error bars is defined in Supplementary Note 2.3. The dashed horizontal line in (a) corresponds to the mean of the values between 275 K and 310 K. Source Data is from Supplementary Table 8.

2.3.1 Branch-point eigenvector

The branch-point eigenvector is defined as the eigenvector of the eigenvalue that goes to 0 as $\tau \to \tau_{\mathrm{BP}}$. For a ring-polymer with N beads on an f-dimensional PES, this is a vector of length Nf. For every bead, the f-dimensional sub-vector of the branch-point eigenvector can be expressed in the basis of the normal modes of the product minimum. Such a projection along the dominant normal modes, as a function of the bead number, is shown in Supplementary Figure 7. The projection of the unit vector along the instanton velocity (i.e., the difference in configuration between consecutive beads) along the same normal modes is also shown, to illustrate the fact that the branch-point eigenvector mostly points along the instanton.

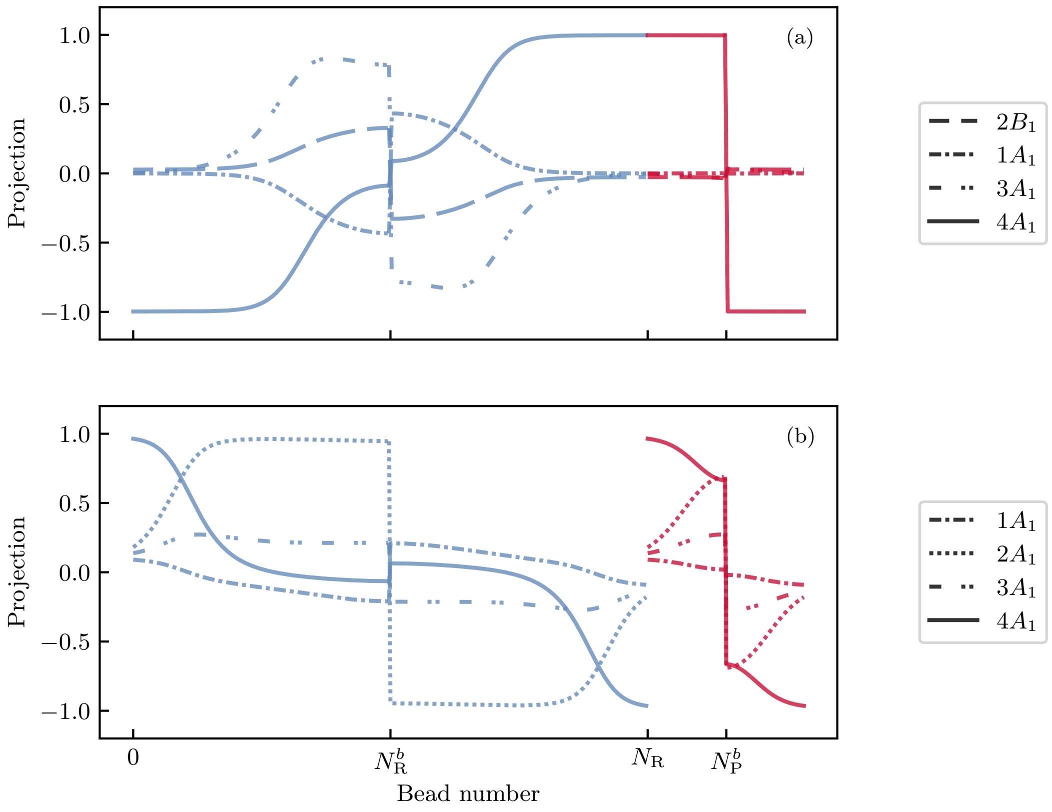

Supplementary Figure 7: Projection of (a) the branch-point eigenvector and (b) the instanton velocity along a selection of normal modes of the reactant minimum, as a function of bead number. The branch-point eigenvector is defined as the eigenvector which corresponds to the eigenvalue of the ring-polymer Hessian that goes to zero at the branch point. All projections were done between unit vectors. Beads 0 and N_{R} correspond to the hopping points $\tilde{x}'$ and $\tilde{x}''$ respectively while the instanton bounces and changes direction at beads $N_{\mathrm{R}}^b = N_{\mathrm{R}}/2$ and $N_{\mathrm{P}}^b = N_{\mathrm{R}} + N_{\mathrm{P}}/2$. The colour denotes whether the bead lies on the reactant (blue) or product (red) PESs. Data for the hessian at the reactant minimum and the mass-weighted branch-point eigenvector is available as a Source Data file.

2.3.2 PCM calculations

Minimum and instanton optimizations were performed by including a PCM in the MCSCF procedure for $O_2 \cdots H_2O$. The basis set and active space remained unchanged at cc-pVDZ and (12,8) respectively. The branch-point instanton and the reactant minimum are depicted in Supplementary Figure 8.

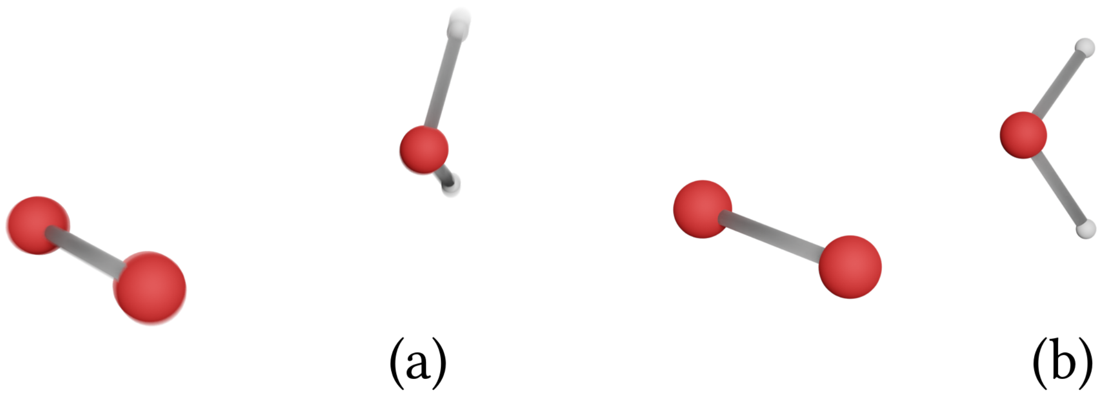

Supplementary Figure 8: The configurations of (a) the instanton and (b) the reactant minimum for $O_2 \cdots H_2O$ with PCM. Geometries are available as Source Data files.

2.4 Equilibrium constants

In order to estimate the equilibrium constant, we use the approximation of a gas-phase reaction but work in the canonical (NVT) ensemble so as to remove the pV contribution as the liquid is almost incompressible. The equilibrium constant is therefore evaluated as

$$K_c \approx \frac{Z_{O_2 \cdots H_2O}}{Z_{O_2} Z_{H_2O}}. \tag{13}$$

The gas-phase partition functions are computed using the standard method of separating into translational, rotational, vibrational and electronic contributions using the harmonic-oscillator and rigid-rotor approximations. Numerical results are presented in Table 9. It can be seen that the equilibrium constants are almost identical for $O_2 \cdots H_2O$ and $O_2 \cdots D_2O$, which implies that nuclear quantum effects are unimportant in K_c and that the kinetic isotope effect can be determined solely from k_2.

Supplementary Table 9: Equilibrium constants for the complex formation K_c as function of temperature, computed using the gas-phase approximation. The formation energy of the complex is $-0.0011\,E_h$. The zero-point energies of $O_2 \cdots H_2O$, $O_2 \cdots D_2O$, H_2O, D_2O and O_2 are $0.0270\,E_h$, $0.0206\,E_h$, $0.0230\,E_h$, $0.0168\,E_h$ and $0.0033\,E_h$, relative to their respective minimum. The concentration of the solvent was obtained from the density data from [3, 4]. For temperatures where the density data was unavailable, data at the closest available temperature was chosen, which was usually within 1 K.

System	T (K)	K_{trans} (L mol^{-1})	K_{rot}	K_{vib}	K_{el}	K_c (L mol^{-1})	[Solvent] (mol L^{-1})	K_c [Solvent]
$O_2 \cdots H_2O$	275	1.796×10^{-5}	5.648	378.973	3.355	0.129	55.520	7.162
	280	1.748×10^{-5}	5.547	415.050	3.283	0.132	55.515	7.337
	290	1.659×10^{-5}	5.356	495.424	3.151	0.139	55.456	7.692
	300	1.577×10^{-5}	5.177	587.767	3.033	0.146	55.347	8.053
	310	1.501×10^{-5}	5.010	693.365	2.926	0.153	55.135	8.413
	320	1.431×10^{-5}	4.854	813.596	2.830	0.160	54.936	8.785
	330	1.367×10^{-5}	4.706	949.926	2.742	0.168	54.706	9.165
	340	1.307×10^{-5}	4.568	1103.919	2.662	0.175	54.356	9.534
	350	1.251×10^{-5}	4.438	1277.234	2.588	0.184	54.064	9.923
	360	1.199×10^{-5}	4.314	1471.633	2.521	0.192	53.750	10.318
$O_2 \cdots D_2O$	280	1.583×10^{-5}	2.431	1044.948	3.283	0.132	55.231	7.291
	290	1.501×10^{-5}	2.348	1246.931	3.151	0.139	55.226	7.649
	300	1.427×10^{-5}	2.269	1478.954	3.033	0.145	55.151	8.011
	310	1.359×10^{-5}	2.196	1744.244	2.926	0.152	54.976	8.372
	320	1.295×10^{-5}	2.127	2046.252	2.830	0.160	54.792	8.743
	330	1.237×10^{-5}	2.063	2388.659	2.742	0.167	54.577	9.121
	340	1.183×10^{-5}	2.002	2775.384	2.662	0.175	54.237	9.489
	350	1.132×10^{-5}	1.945	3210.589	2.588	0.183	53.953	9.876
	360	1.086×10^{-5}	1.891	3698.692	2.521	0.191	53.643	10.268

2.5 Temperature dependence

In Ref. [4], the temperature dependence of the nonradiative rate constants in both H_2O and D_2O were measured experimentally. The PaAD model was constructed in Ref. [5] to fit the data using 3 parameters for the H_2O and D_2O cases separately.

We wish to demonstrate that our new theory can capture the trends observed in experiment. However, it would be naïve to assume that our theory can predict the experimental rates quantitatively due to the approximations made, such as those within the electronic structure. Even more importantly, we would require a precise knowledge of the equilibrium constant K_c, for which we have only made a very rough order-of-magnitude estimate based on a gas-phase picture.

We therefore set ourselves a more achievable goal of fitting the equilibrium constant to demonstrate that our calculations are at least not inconsistent with the experimental data. In the main text we make the stronger claim that our calculations can accurately predict the kinetic isotope effect from first principles due to the fact that the equilibrium constant cancels in the ratio of overall rate constants.

First, we define

$$c^{\ominus} K_c = e^{-\Delta_c G^{\ominus}/RT} = e^{-\Delta_c H^{\ominus}/RT + \Delta_c S^{\ominus}/R}, \tag{14}$$

where $R = 8.31\,\mathrm{J\,K^{-1}\,mol^{-1}}$ is the molar gas constant and $c^{\ominus} = 1\,\mathrm{M} = 1\,\mathrm{mol\,L^{-1}}$ is a reference concentration. Then by fitting (by eye), we found the parameters $\Delta_c H^{\ominus}/R = 190\,\mathrm{K}$ and $\Delta_c S^{\ominus}/R = -0.75$ (or in standard units $\Delta_c H^{\ominus} = 1.58\,\mathrm{kJ\,mol^{-1}}$ and $\Delta_c S^{\ominus} = -6.24\,\mathrm{J\,K^{-1}\,mol^{-1}}$). These values are certainly physically reasonable. In particular, they imply that the complex has a lower entropy, as it is less disordered than the ordinary liquid, but a higher enthalpy, as it disrupts the hydrogen-bonding network of water. Note that according to these definitions, $\Delta S^{\ominus}$ is the entropy change for complexation with a pre-determined water molecule. In order to obtain the overall probability that an $^1O_2$ molecule will be found as part of a complex (regardless of which water molecule it chooses), one also needs to take into account the fact that there are a large number of H_2O molecules. This is accounted for by $K_c[H_2O] \approx 55 c^{\ominus} K_c$, which appears in k_{eff}, but not in k_{nr}.

Note that here, we have assumed classical statistical mechanics is sufficient to describe K_c, which implies that it will be agnostic to the isotopes. In reality there will probably be small differences between H_2O and D_2O due to quantum nuclear effects, but these are expected to be much smaller than the kinetic isotope effects due to the tunnelling process. In practice, the values of the parameters we have fitted also incorporates an overall shift to the rate which may account for some of the discrepancies of the electronic-structure methods.

We find that for $T \geq 330\,\mathrm{K}$, a second (and sometimes a third) branch point encroaches on the first branch point, which invalidates our theory as it currently stands. Although it would be possible to extend the theory to treat multiple branch points, it is anyway unlikely that the approximate $\Delta_c H^{\ominus}$ and $\Delta_c S^{\ominus}$ parameters remain valid at higher temperatures, where it is expected that the hydrogen-bonding network is significantly modified (as pointed out in Ref. [6]). For these reasons, only the low-temperature calculated results are presented in the plot.

Comparing the resulting rates to the experimental measurements gives a good agreement, as shown in Fig. 9. Note that we have achieved this agreement for both H_2O and D_2O using only two fitting parameters in total, whereas Ref. [5] required six separate parameters. This demonstrates that our results are at least not inconsistent with previous work and lends further weight to our claims that the mechanism proceeds in the way we have described.

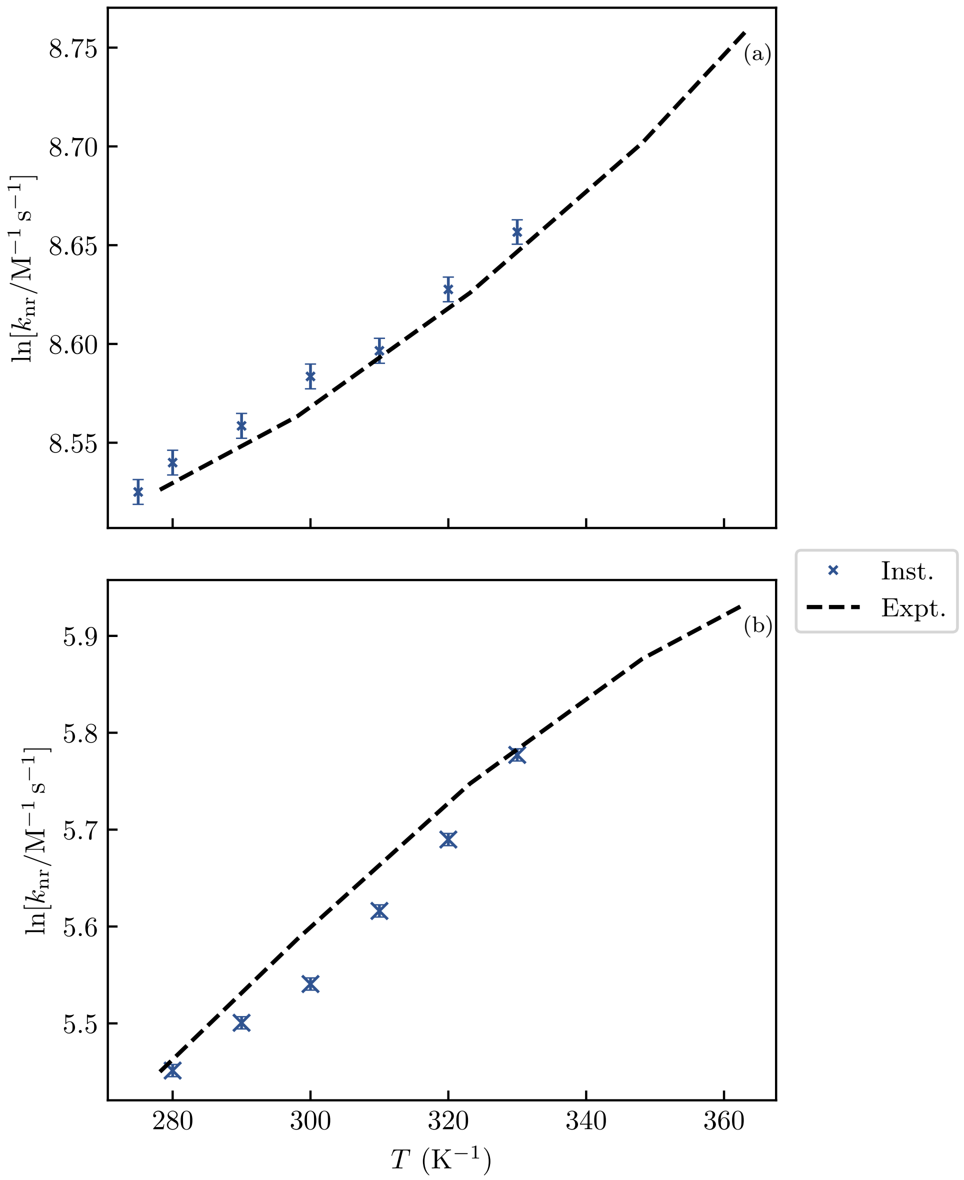

Supplementary Figure 9: Second-order nonradiative rate constants $k_{\rm nr}$ as a function of temperature for (a) $O_2\cdots H_2O$ and (b) $O_2\cdots D_2O$. To obtain $k_{\rm nr}$ from the first-principles instanton results, we used $k_{\rm nr} = k_{\rm BP}K_{\rm c}$, where the equilibrium constant was fitted with two parameters as explained in Supplementary Note 2.5. Theoretical error bars for $k_{\rm nr}$ are estimated as in Supplementary Figure 6 and then multiplied by $K_{\rm c}$. The experimental data was obtained from Ref. [4]. Note that the experimental error bars (not shown) for $O_2\cdots H_2O$ on this log scale are on the order of ± 0.05. Source data for the instanton calculations are from Supplementary Table 8 and the equilibrium constants are from Supplementary Table 9.

Supplementary references

[1] I. M. Ansari, E. R. Heller, G. Trenins, and J. O. Richardson, “Instanton theory for Fermi’s golden rule and beyond,” *Phil. Trans. R. Soc. A.*, vol. 380, p. 20200378, 2022.

[2] J. O. Richardson, “Ring-polymer instanton theory,” *Int. Rev. Phys. Chem.*, vol. 37, pp. 171–216, 2018.

[3] E. Lemmon, M. Huber, and M. McLinden, “Nist standard reference database 23: Reference fluid thermodynamic and transport properties-refprop, version 9.1,” 2013-05-07 2013.

[4] M. Bregnhøj, M. Westberg, F. Jensen, and P. R. Ogilby, “Solvent-dependent singlet oxygen lifetimes: temperature effects implicate tunneling and charge-transfer interactions,” *Phys. Chem. Chem. Phys.*, vol. 18, pp. 22946–22961, 2016.

[5] F. Thorning, P. Henke, and P. R. Ogilby, “Perturbed and activated decay: The lifetime of singlet oxygen in liquid organic solvents,” *J. Am. Chem. Soc.*, vol. 144, no. 24, pp. 10902–10911, 2022.

[6] F. Thorning, F. Jensen, and P. R. Ogilby, “Modeling the effect of solvents on nonradiative singlet oxygen deactivation: Going beyond weak coupling in intermolecular electronic-to-vibrational energy transfer,” *J. Phys. Chem. B*, vol. 124, no. 11, pp. 2245–2254, 2020.
