# Supplementary material for: Heavy-atom tunnelling in singlet oxygen deactivation predicted by instanton theory with branch-point singularities
Source: Nat Commun. 2024 May 21;15:4335. doi: 10.1038/s41467-024-48463-2 (PMC11522392; doi:10.1038/s41467-024-48463-2)
Supplement: Supplementary file 3 — Description of Additional Supplementary Files [file 41467_2024_48463_MOESM3_ESM.pdf]

Description of Additional Supplementary Files:

Supplementary Data 1: The folder ’instantons’ contains instanton data: O2-H2O.dat: data file from O2-H2O fixed-tau calculations at 300 K. O2-D2O.dat: data file from O2-D2O fixed-tau calculations at 300 K. 18O18O-H2O.dat: data file from 18O18O-H2O fixed-tau calculations at 300 K.

branch-point/O2-H2O.xml: xml file with all the data about the branch-point instanton of O2-H2O at 300 K, including the trajectory, its action, potentials, gradients and hessians along the trajectory. branch-point/O2-H2O.xyz: xyz file of the branch-point instanton trajectory for O2-H2O at 300 K. branch-point/O2-D2O.xml: xml file with all the data about the branch-point instanton of O2-D2O at 300 K, including the trajectory, its action, potentials, gradients and hessians along the trajectory. branch-point/O2-D2O.xyz: xyz file of the branch-point instanton trajectory for O2-D2O at 300 K. branch-point/18O18O-H2O.xml: xml file with all the data about the branch-point instanton of 18O18O-H2O at 300 K, including the trajectory, its action, potentials, gradients and hessians along the trajectory. branch-point/18O18O-H2O.xyz: xyz file of the branch-point instanton trajectory for 18O18O-H2O at 300 K. branch-point/O2-H2O_pcm.xml: xml file with all the data about the branch-point instanton of O2-H2O at 300 K using PCM solvation, including the trajectory, its action, potentials, gradients and hessians along the trajectory. branch-point/O2-H2O_pcm.xyz: xyz file of the branch-point instanton trajectory for O2-H2O at 300 K using PCM solvation. branch-point/branchpoint_eigvec_H2O.dat: mass-weighted branch-point eigenvector of O2-H2O at 300 K. This is a textfile with 512 rows,corresponding to each bead and 15 columns, corresponding to the 15 cartesian degrees of freedom, ordered by atoms. The order of the atoms is [O, O, O, H, H]. branch-point/instplot_H2O0.dat: reactant potentials (MCSCF) along the mass-weighted path length of the O2-H2O branch-point instanton at 300 K. branch-point/instplot_H2O1.dat: product potentials (MCSCF) along the mass-weighted path length of the O2-H2O branch-point instanton at 300 K. branch-point/instplot_mrmp2_H2O0.dat: reactant potentials (MRMP2) along the mass-weighted path length of the O2-H2O branch-point instanton at 300 K. branch-point/instplot_mrmp2_H2O1.dat: product potentials (MRMP2) along the mass-weighted path length of the O2-H2O branch-point instanton at 300 K.

The folder ’minima’ contains data for the minima: min_product.xml: xml file with all data at the product minimum, including geometry, gradient, hessian and masses of the atoms in O2-H2O. min_product.xyz: xyz file of the product minimum. min_reactant.xml: xml file with all data at the reactant minimum, including geometry, gradient, hessian and masses of of the atoms in O2-H2O. min_reactant.xyz: xyz file of the reactant minimum. min_reactant_pcm.xml: xml file with all data at the reactant minimum using PCM solvation, including geometry, gradient, hessian and masses of of the atoms in O2-H2O. min_reactant_pcm.xyz: xyz file of the reactant minimum using PCM solvation.

The folder ’saddle_point’ contains data for the product sadle point: saddle_product.xml: xml file with all data at the product saddle point, including geometry, gradient, hessian and mass. saddle_product.xyz: xyz file of the product saddle point.

The folder 'mecp' contains data for the MECP: mecp.xml: xml file with all data at the MECP, including geometry, gradient, hessian, lagrange multiplier and masses of the atoms in O2-H2O. mecp.xyz: xyz file of the MECP.
